# Effect of thin porous ceramic coatings on implant stability: a comparative study of GB14 and β-TCP with and without Cu

**DOI:** 10.1007/s10856-026-07013-6

**Published:** 2026-02-17

**Authors:** Mihail Genchev, Coralie Nagels, Hagen Schmal, Eva Johanna Kubosch, Maria Carolina Lanzino, Andreas Killinger, Sofia Dembski, Anika Höppel, Jakob Neubauer, Michael Seidenstuecker

**Affiliations:** 1 G.E.R.N. Research Center for Tissue Replacement, Regeneration & Neogenesis, Department of Orthopedics and Trauma Surgery, Medical Center-Albert-Ludwigs-University of Freiburg, Faculty of Medicine, Albert-Ludwigs-University of Freiburg, Freiburg, Germany; 2 Department of Orthopedics and Trauma Surgery, Medical Center-Albert-Ludwigs-University of Freiburg, Faculty of Medicine, Albert-Ludwigs-University of Freiburg, Freiburg, Germany; 3 Department of Orthopedic Surgery and Traumatology, Odense University Hospital, Odense, Denmark; 4 Institute for Manufacturing Technologies of Ceramic Components and Composites (IFKB), University of Stuttgart, Stuttgart, Germany; 5 Fraunhofer Institute for Silicate Research ISC, Würzburg, Germany; 6 Department for Functional Materials in Medicine and Dentistry, University of Würzburg, Würzburg, Germany; 7 Department of Diagnostic and Interventional Radiology, Medical Center-Albert-Ludwigs-University of Freiburg, Faculty of Medicine, Albert-Ludwigs-University of Freiburg, Freiburg, Germany

## Abstract

The present study investigates the effect of thin porous ceramic coatings on implant stability, focusing on two materials: a calcium alkali orthophosphate (GB14, Ca_2_KNa(PO_4_)_2_) and β-tricalcium phosphate (β-TCP), with and without copper (Cu) incorporation. The coatings were applied to titanium implant surfaces (CP Ti, grade 2) and characterized for porosity and microstructure. The in vivo performance of the material is assessed in a New Zealand White rabbit model. Following defined healing periods, biomechanical push-out testing were performed. The results of β-TCP/Cu for cancellous bone show that Cu-doped coatings exhibit significantly improved bone integration compared to their Cu-free counterparts. The enhanced fixation is attributed to the bioactive and potential antibacterial properties of copper, which may stimulate osteogenesis and the presence of supraparticles in the Cu samples. Furthermore, the incorporation of β -TCP supraparticles into the ceramic matrix increases overall coating porosity, facilitating deeper bone ingrowth and improved mechanical interlocking. This structural change results in improved osseointegration compared to less porous coatings. This structural change results in improved osseointegration compared to less porous coatings. The results of this study demonstrate that combining copper incorporation with enhanced porosity through supraparticles can improve implant stability by shortening the time required for the transition from primary to secondary stability. This approach offers a promising strategy for optimizing surface design in orthopedic and dental implants.

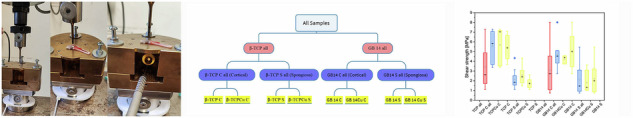

## Introduction

Joint replacement surgery is a critical intervention to restoring mobility and relieving pain in patients with degenerative joint disease [1, 2]. Despite advances in surgical techniques and implant materials, complications such as aseptic loosening and periprosthetic joint infection (PJI) continue to occur, often requiring revision surgery [3]. The American Joint Replacement Registry (AJRR) Annual Report covering the period from 2012 to 2023 indicates that revision procedures accounted for 9.4% of all hip arthroplasties and 8.6% of all knee arthroplasties. The predominant reasons for revision were periprosthetic joint infection (PJI) or infection-induced inflammatory reactions. These reasons accounted for 21.4% of hip revisions and 33.1% of knee revisions. Aseptic or mechanical complications, such as implant loosening or instability, represented 32.0% and 38.6% of hip and knee revisions, respectively. The main cause of implant-associated infections is *Staphylococcus aureus* [4]. In response to these clinical challenges, a novel implant surface coating has been developed, to address the four most prevalent causes of revision surgery. This multifunctional coating incorporates copper (Cu), a transition metal known for its antibacterial properties [5]. By generating reactive oxygen species that disrupt membrane integrity, interfere with ATP production, and inhibit DNA replication, Cu-doped coatings may reduce the incidence of PJI [6, 7]. Furthermore, the coating employs β-tricalcium phosphate (β-TCP) as the osteoconductive matrix, selected for its superior resorption kinetics and osseointegration potential compared to hydroxyapatite (HA) [8]. The enhanced biodegradability of β-TCP facilitates rapid bone remodeling and stable implant fixation, which potentially lowers the risk of aseptic loosening and mechanical failure [9]. This multifunctional approach, integrating antimicrobial efficacy with improved osseointegration, represents a targeted strategy to reduce both infection-related and mechanical complications in total joint arthroplasty. These complications not only affect patient outcomes, but also place a significant economic burden on healthcare systems [10–12]. To improve implant longevity and integration, surface modifications of orthopedic implants have attracted considerable attention. Among these, calcium phosphate (CaP)-based coatings, particularly HA, have been extensively studied due to their chemical similarity to the mineral component of bone, which promotes osteoconductivity and facilitates bone-to-implant integration [13]. However, HA coatings have limitations, including brittleness and the potential for delamination under mechanical stress [14–16]. Alternative CaP materials such as β-TCP [17, 18] and GB14 [19] have emerged as promising candidates. β-TCP offers greater solubility than HA, potentially enhancing resorption and subsequent bone regeneration [20]. GB14, developed by the German Federal Institute for Materials Research and Testing (BAM), has rapid resorption rates due to its high alkali content, which may further support bone healing processes [21, 22]. The method by which these coatings were applied has a significant impact on their performance. High Velocity Suspension Flame Spraying (HVSFS) has emerged as a technique capable of producing thin, uniform coatings with controlled porosity and crystallinity [23, 24]. Such properties are critical for achieving a balance between mechanical stability and biological activity. Previous studies have demonstrated the feasibility of using HVSFS to apply HA [25], β-TCP [26], and GB14 [27] coatings. Moreover, the preliminary study demonstrated that β-TCP and GB14 exhibited superior biocompatibility compared to the other biomaterials tested, HA and BG, in terms of our thin coatings [8, 9]. In order to reduce the time required for achieving primary and secondary stability and to promote accelerated bone tissue ingrowth, spray dried β-TCP supraparticles (80 ± 40 µm) [28], composed of nanoparticles (nanorods 28 ± 15 nm × 6 ± 4 nm) [28], were incorporated into the coating during the HVSFS process, in addition to the coating material. This process resulted in the formation of a more porous coating. In addition to promoting osseointegration, implant coatings and the supraparticles can also serve as carriers for antimicrobial agents to prevent PJI. The incorporation of metal ions such as silver and Cu into CaP coatings has demonstrated potential antibacterial activity. In particular, Cu at appropriate concentrations has been shown to be effective against common pathogens such as Staphylococcus aureus while maintaining cytocompatibility [7, 25, 26]. Mechanistically, copper primarily exerts its antibacterial activity by generating reactive oxygen species (ROS), leading to oxidative damage of bacterial cell membranes. In addition, copper ions interfere with essential metabolic pathways, disrupt protein function, and impair DNA replication, collectively resulting in bacterial inactivation. Building on these findings, this study aims to biomechanically evaluate the in vivo performance of HVSFS-coated β-TCP and GB14 coatings with and without Cu-doping. We hypothesize that these coatings will enhance bone integration and exhibit antibacterial properties (against *Staphylococcus aureus*), thereby addressing two critical challenges in joint arthroplasty. This study represents the final stage, in the form of an animal experiment, of all previous studies on biocompatibility [7, 26], antimicrobial efficacy [26, 29], and release of active substances [30] from the new thin coating. The focus here is on biomechanics.

## Materials and methods

### Suspensions and coating deposition

To fabricate thin porous coatings on Ti rods (TiAl6Nb7 (CP Ti, grade 2), length 240 mm, diameter = 4 mm; DePuy Synthes, Johnson and Johnson, New Brunswick, New Jersey, USA), four different suspensions were prepared. The raw powder content of β-TCP (Ca_3_(PO_4_)_2_, β-TCP, 97.2%, Chemical Specialist Budenheim, Budenheim, Germany) and GB14 (Ca_2_KNa(PO_4_)_2_, BAM, Berlin, Germany) was set to 5 wt.%. Deionized (DI) water served as the suspension medium. To stabilize the suspensions, two different stabilizing agents were employed: 2 wt.% of a hydrocolloid (Zschimmer & Schwarz, Lahnstein, Germany) and 3 wt.% of the solid content of a phosphonate-based dispersion agent (Zschimmer & Schwarz, Lahnstein, Germany). For the Cu-doped coatings, 0.5 wt.% of Cu-doped supraparticles (containing 5 wt.% Cu) were incorporated into the previously described suspension in a second processing step. During suspension preparation, the powders or supraparticles were gradually added to deionized water containing the stabilizers under continuous stirring. A modified Top-Gun-G system (GTV Verschleißschutz, Luckenbach, Germany) was used for coating deposition via HVSFS. The spraying torch was mounted on a six-axis robot, which executed a controlled meander movement across the Ti rods. Spraying distances were 120 mm for β-TCP and 140 mm for GB14 coatings (with and without Cu-doped supraparticles). These parameters were optimized in previous studies to ensure consistent coating thickness, porosity, and surface roughness [25, 26]. Prior to spraying, the Ti rods were engraved using a CNC lathe to facilitate their subsequent segmentation into 16 mm long samples for implantation. The full-length (240 mm) rods were grit-blasted with F60 corundum at 4 bar, which increased their roughness to the end value of Ra = 2.68 ± 0.24 µm, Rz = 17.58 ± 1.07 µm, then cleaned with acetone in an ultrasonic bath for 1 min (RK 510, SONOREX, BANDELIN electronic GmbH & Co. KG, Berlin, Germany) and weighed. To ensure uniform coating deposition, the rods were mounted on a lathe. The rotation speed was set to 1250 rpm, and the robot traverse speed to 250 mm/s. Ethylene (C₂H₄) and oxygen (O₂) were used as combustion gases, with flow rates of 70 slpm and 125 slpm, respectively. A 22-8-135 combustion chamber (22 mm nozzle length, 8 mm nozzle diameter, total length 135 mm) was employed. During spraying, the rods were cooled from the rear side to minimize thermal load on the specimens. For the β-TCP samples, 20 total passes were applied per rod, whereas the β-TCP/Cu, GB14, and GB14/Cu coatings received 24 passes each. β-TCP had a higher coating efficiency, which meant that fewer cycles were required to achieve a similar layer thickness. This could be due to the coarser β-TCP/Cu supraparticles added in the suspension, making this more unstable.

Each material system was coated on a full 240 mm rod, subsequently segmented into 11 shorter rods. Eight samples of each were sent to Freiburg for implantation, while the remainder were used for coating characterization. Due to the CNC engraving process, one segment measured 15 mm in length and was specifically used for X-ray diffraction (XRD) and surface roughness measurements.

### Coating characterization

#### SEM

For the characterization of the coatings, details of the microstructures were observed using a field-emission scanning electron microscope (SEM) S-800 (Hitachi High-Technologies Corporation, Tokyo, Japan) equipped with a sensor for backscattered electrons (BEC). Cross-section samples were sputtered with carbon before SEM examination. SEM images were additionally utilized to evaluate the coating porosity using image processing software (ImageJ 1.47 v) with a contrast threshold for analysis. To do this, the images were first converted to 8-bit format and then segmented using a uniform thresholding method to separate pores (dark areas) from the base material. The percentage pore area was then calculated by binary analysis (Analyze Particles). We performed and averaged the analysis on three SEM images at a magnification of x1400, 10 kV.

#### XRD

The phase composition of the coatings was analyzed by X-ray diffraction (XRD: X’Pert PRO, PANAlytical, Almelo, The Netherlands) using Cu–Kα radiation (λ = 0.1541 nm). The diffraction patterns were collected in the 20°–70° 2θ range (step size: 0.02°; scan rate: 5 s/step). The used sample for measurement was the longer rod (length = 15 mm).

#### Surface roughness

Coating roughness values R_a_ and R_z_ were investigated by tactile measurement with Mahr Perthometer (Mahr GmbH, Göttingen, Germany). The measurement on a longer rod of 15 mm was performed with a measurement length of 14 mm and five single measurements according to DIN EN ISO 3274. For each coating, the values and standard deviation were determined taking the average values.

#### Optical microscopy

Coating microstructures were analyzed through an optical microscope MeF4M (Leica GmbH, Wetzlar, Germany) in bright field. Pictures were taken and analyzed by the Aquinto a4i analysis software (Olympus Europa SE & Co. KG, Hamburg, Germany). Coating thicknesses were characterized according to DIN EN ISO 1463:2021-08 by measuring fifteen single coating thickness values and respectively calculating average value and standard deviation.

### Animal Model

The animal model used in this study including implantation procedure, harvesting and preparation was reported previously [17]. 12 New Zealand White rabbits (Charles River Laboratories, Écully, France) were used in the study. Each rabbit was at least 26 weeks old to ensure closure of the epiphysis. The mean weight of each animal was 3.75 ± 0.16 kg. Before and after cylinder implantation, the rabbits were allowed to bear full weight on their legs. The experiment was approved by the **Regional Council of Freiburg (Reference number: G-23/079)** and carried out in accordance with **§8 TierSchG, Directive 2010/63/EU** and **ISO EN 10993-2:2023-02** animal welfare requirements [31]. The animals were divided into two groups: GB14 and β-TCP. These two groups were further subdivided into with/without Cu. This means that the animals received an implant with Cu in their left knee and a Cu free implant in their right knee. This resulted in a final total of four groups of six animals. According to the previous power calculation by the biostatistics office of the University of Freiburg, 5 animals per group were necessary for sufficient power.

#### Implantation of coated implants

The 12 New Zealand White rabbits (Charles River, Écully, France) were divided into 2 groups of 6 animals each (β-TCP, GB14 group with/without Cu), with a respective holding time of 24 weeks after the surgical procedure. The surgery was performed under sterile conditions. For analgosedative premedication, 35.0 mg/kg ketamine (Serumwerke Bernburg, Bernburg, Germany) and 0.1–0.25 mg/kg medetomidine (Domitor, Orion pharma, Espoo, Finland) were injected intramuscularly. Analgesia was achieved by injecting 4.0 mg/kg of carprofen (Rimadyl, Zoetis Inc., Parsippany, NJ, USA) subcutaneously in combination with a single dose of 30.0 mg/kg of metamizole (Novalgin, A. Natter & Cie GmbH, Frankfurt, Germany) intramuscularly. After analgosedation, the animals’ eyes were treated with Bepanthen eye ointment (Bayer Vital GmbH, Leverkusen, Germany) as a precaution. To maintain anesthesia, 0.5–2.5% isoflurane (CP Pharma, Burgdorf, Germany) in an oxygen-air mixture (FiO_2_ > 0.4) was individually adjusted. Perioperative analgesia was achieved with 0.01–0.02 mg/kg/h of fentanyl (Hameln Pharma, Hameln, Germany) intravenous. Lidocaine (B. Braun SE, Melsungen, Germany) was administered at the surgical sites for local anesthesia. Surgery was performed in the supine position. The animals were shaved in the surgical area, and the skin was sterilized with pvp iodine (Braunoderm®, B. Braun SE, Melsungen, Germany). The pins were implanted in the distal femoral condyle, accessing from the lateral on both sides, with the pin with Cu doting on the left and the pin without Cu doting on the right. After palpating the joint space of the knee joint and the epicondyle lateralis femoris, an incision of approximately 2 cm in length was made proximal to the joint space above the epicondyle, parallel to the long axis of the femur. After preparing the surgical area down to the bony structures, the distal femoral condyle was pre-drilled using a 4.0 mm spiral drill. The pin was then driven into the prepared drill hole using an impactor (ActivaPin®, B-IP-2000 Applicator 4.0, bioretec, Tampere, Finland). The wounds were then sutured, and the rabbits received a single dose of 0.025 mg/kg buprenorphine (Temgesic, Eumedica Pharmaceuticals GmbH, Lörrach, Germany) subcutaneously. To assess bone formation, animals were also injected subcutaneously with 4 different fluorescent dyes. Rolitetracycline, xylenol orange (both, Sigma Aldrich, now Merck, Darmstadt, Germany) calcein, alizarin complexone (both, Carl Roth GmbH + Co. KG, Karlsruhe, Germany), were dissolved in a 2% aqueous NaHCO_3_ (Sigma Aldrich, now Merck, Darmstadt, Germany) solution, so that 1 ml of injection solution could be administered per animal [32]. Each dye is injected only once. Dyes are administered 2, 4, 6, and 8 weeks after surgery. The animals were euthanized after 24 weeks. After anesthesia with 20.0 mg/kg ketamine and 0.3 mg/kg midazolam subcutaneously, euthanasia was induced by the subsequent administration of 5.0-10.0 mg/kg propofol (Fresenius Kabi, Bad Homburg, Germany) and 2.0 mmol/kg KCl (Fresenius Kabi, Bad Homburg, Germany) intravenously.

#### Implant harvesting and preparation

**Fig. 1:**
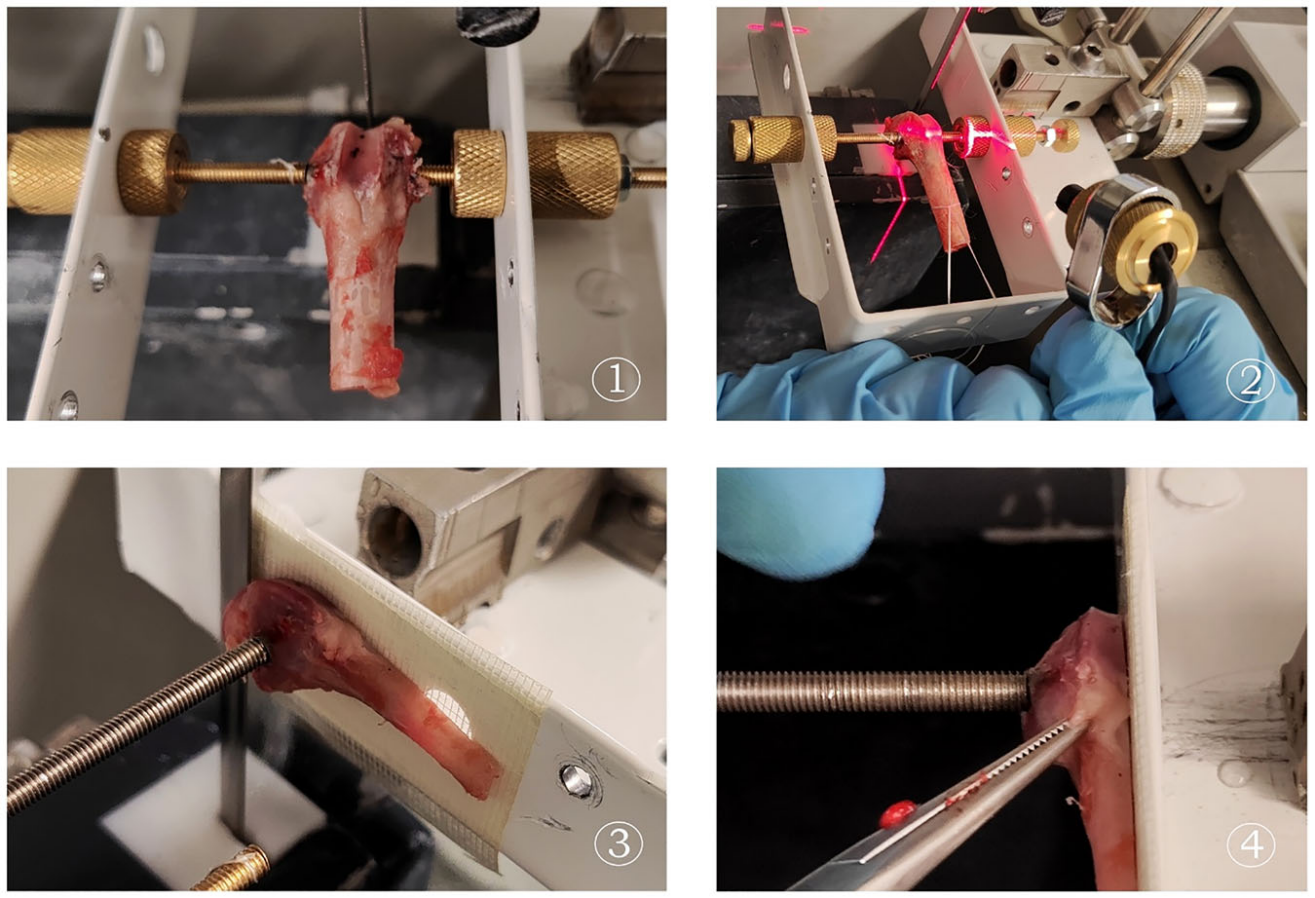
Example images of our holder, which can be used to saw the condyles at a 90° angle to the implant: (1) Position the condyle so that it can be sawed centrally at a 90° angle to the implant; (2) Laser-precise determination of the sawing position; (3, 4) Half condyles fixed at the edge for sawing the 2 mm thick discs

At the specified time interval of 24 weeks, the rabbits were euthanized as previous described. The femurs of the rabbits were dissected, the distal third was removed, and the knee was disarticulated and placed in a solution of 70% ethanol for preservation. Each implant-containing metaphysis was then cut in half using a diamond band saw (Walther Messmer GmbH, Oststeinbeck, Germany). A proprietary holder was used that could be attached directly to the implants and cut the specimens at a 90° angle to the implant to avoid shear forces during the push-out test (Fig. 1). The lateral half of the specimens was used for the push-out tests. For this purpose, they were cut into 2 mm thick slices for standardized push-out tests.

### Push-out Tests

**Fig. 2:**
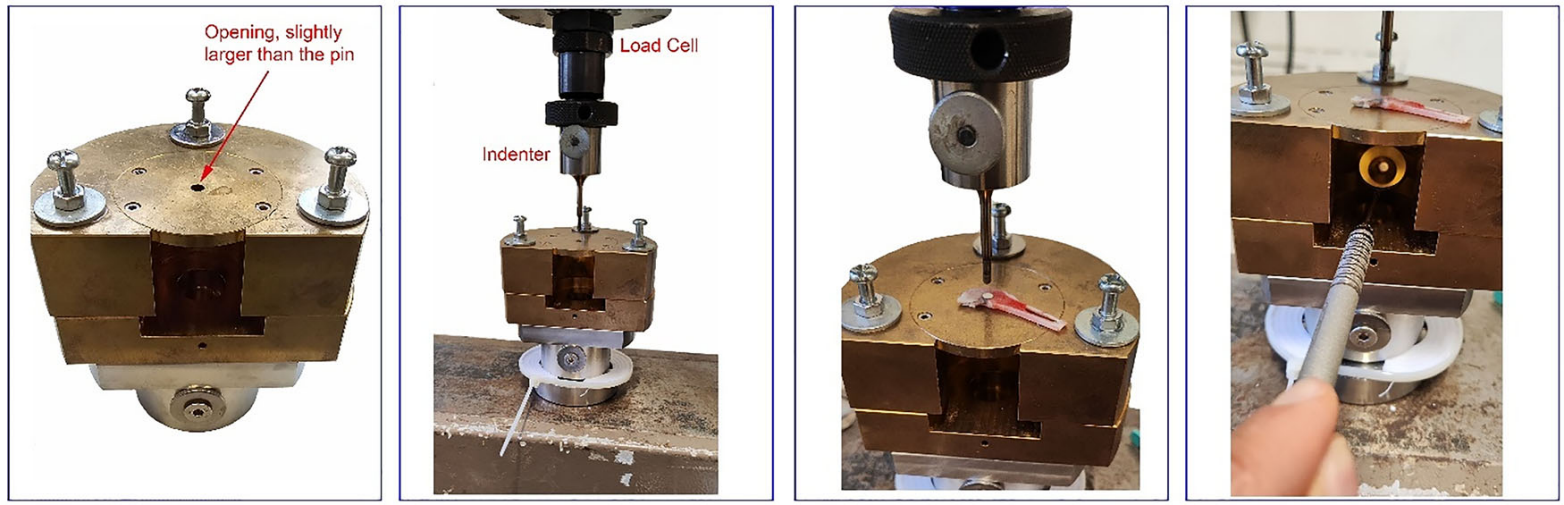
Schematic representation of the push-out-test conditions: by using a mouth mirror, the exact centering of the implant above the intended drilling site can be checked to ensure that it does not tilt during the pushout process

Bone ingrowth into the newly developed thin porous layers was evaluated using push-out tests performed on a universal testing machine (Zwick Zmart, ZwickRoell GmbH & Co. KG, Ulm, Germany) equipped with a 250 N load cell. In order to remain comparable, the push-out test was carried out according to a previously published protocol [9]. Each bone-implant disc was placed on a custom-designed support table with a circular opening slightly larger in diameter than the implant itself to ensure that the implant was centered over the opening. The load was applied using a cylindrical indenter aligned centrally above the implant within the bone disc. Accurate positioning was achieved using a custom-made telescopic needle attached to the indenter. A preload of 1 N was applied to initiate the test. Once this preload was reached, the indenter was advanced at a constant rate of 1 mm/min. Throughout the test, the applied force was recorded in relation to the displacement of the indenter and a force-displacement curve was generated using the TestXpert II software (ZwickRoell GmbH & Co. KG, Ulm, Germany). The test was manually stopped when interfacial shear failure was detected, indicated by a sudden drop in force and visually confirmed by the relative movement of the implant in the surrounding bone. Interfacial shear strength was calculated by dividing the maximum recorded force by the lateral surface area of the implant. Representative photographs of the test setup, bone-implant specimen, and push-out procedure are shown in Fig. 2.

### CT-analysis of the implant position in the bone

**Fig. 3:**
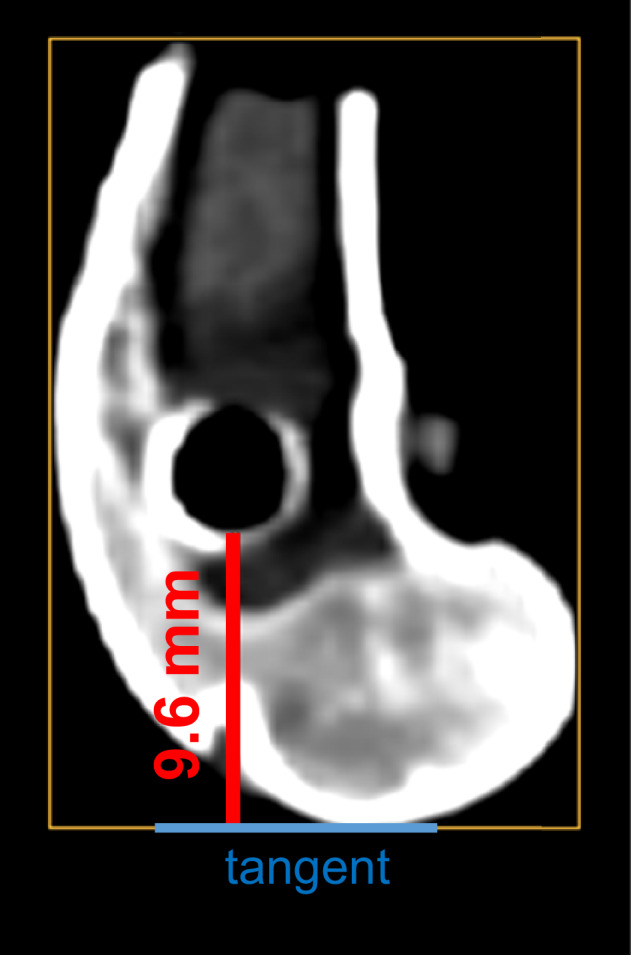
An illustrative case demonstrating the spatial relationship between the prior implant site and the most distal aspect of the femoral condyles, with particular emphasis on the linear distance separating these anatomical landmarks

To evaluate the spatial positioning of the displaced implant following the push-out test, computed tomography (CT) scans were obtained from the sectioned bone samples. The CT scanner NAEOTOM Alpha (Siemens Healthineers AG, Forchheim, Germany) with the following settings was used: a tube current of 120 kV, a matrix size of 1024 × 1024, a slice thickness of 0.2 mm, and a FOV of 150 × 150 mm. These scans were analyzed to determine the relative location of the implant with respect to the most distal aspect of the femoral condyles (Fig. 3). To do this, the CT data set was opened in DeepUnity Diagnost (Dedalus Healthcare, Bonn, Germany), a tangent was inserted at the condyle, and its distance from the drill hole was measured. This approach enabled a detailed assessment of the implant trajectory and its final position post-displacement. Prior studies have demonstrated an inverse relationship between shear strength and the distance of the implant from the distal extremity of the bone, indicating that increased displacement proximally corresponds with reduced mechanical resistance at the bone-implant interface [33].

### Statistical analysis

Data are presented as mean ± standard deviation. For all groups, a one-way ANOVA with multiple comparisons using the Tukey post hoc test was performed at a significance level of 0.05 in each time interval. Origin 2023 Professional SR1 (OriginLab, Northampton, MA, USA) was used for statistical calculations.

## Results

### Coating characterization

**Fig. 4:**
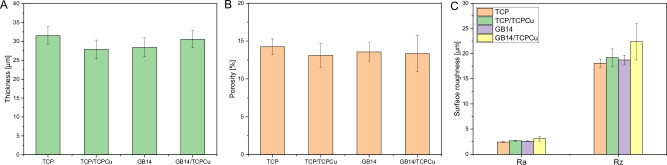
Coating characteristics of the investigated samples. Overview of coating parameters: **A** thickness; **B** porosity; and **C** roughness. (*n* = 3)

The coatings on rods showed a thickness of 29.6 ± 1.7 µm and similar porosity of 13.6 ± 0.6%. The surface roughness Ra was between 2.7 ± 0.6 µm (Fig. 4). In general, coatings with integrated supraparticles showed slightly higher roughness results. A detailed analysis of coatings microstructure with these material systems can be found in previous studies [26, 27].

**Fig. 5:**
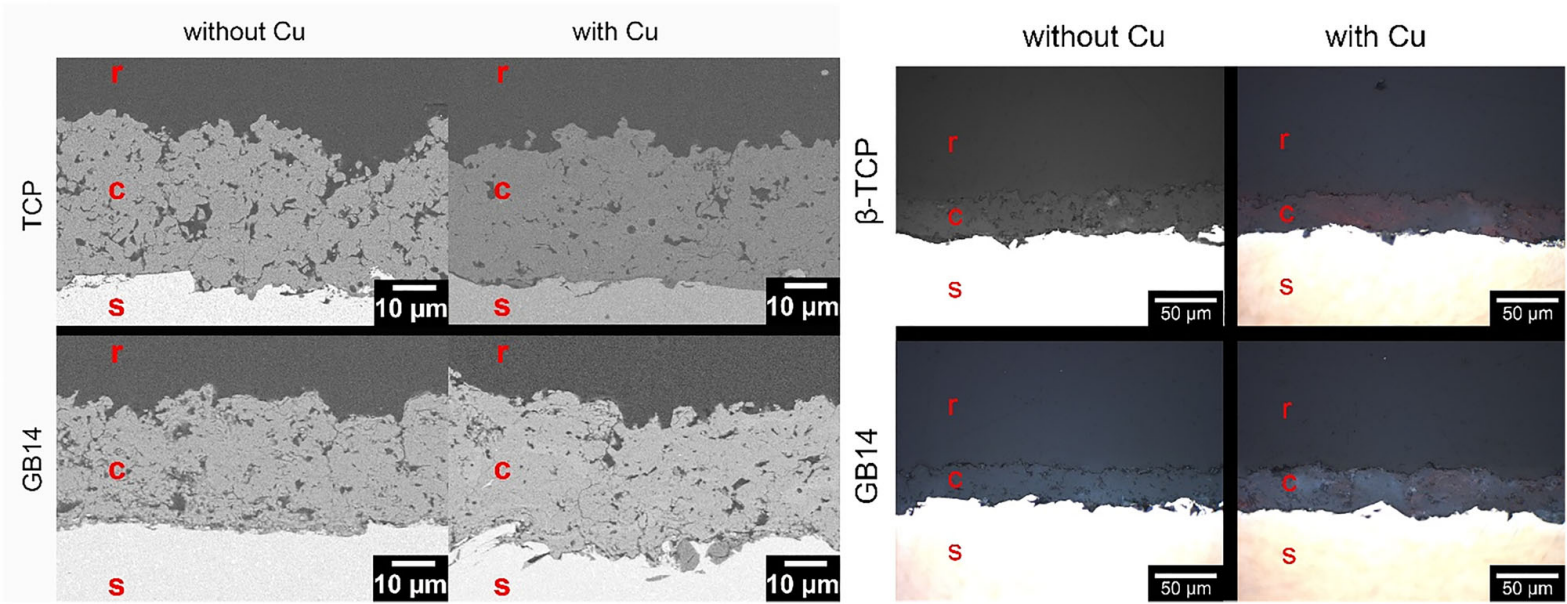
Examples of SEM sections (left) and optical microscopy sections (right) for the different samples: r…resin; c…coating; s…substrate

As illustrated in Fig. 5, a variety of coatings were utilized in this project. It was observed that all layer thicknesses were within the range of 30 µm. In addition, all coatings had a porosity of at least 13% due to the use of supraparticles.

### XRD

**Fig. 6:**
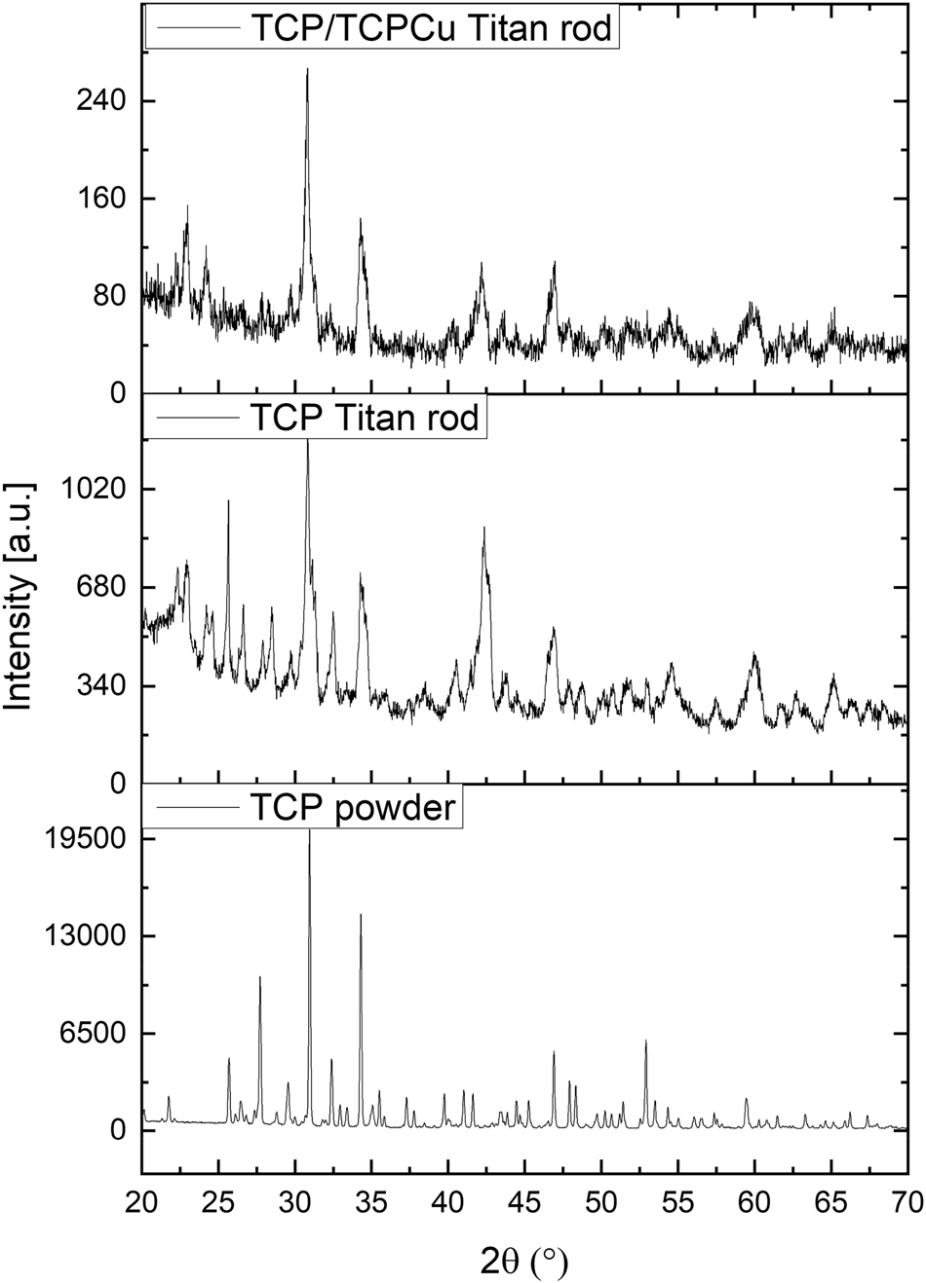
XRD pattern of β-TCP powder and coated Ti rods

The tested β-TCP showed a phase purity of 99.5% with traces of CPP in the Rietveld refinement analysis (Profex 5.2.2.). No influence of the copper in the coating compared to a coating without copper could be detected in the XRD pattern (Fig. 6).

### Push-out tests

**Fig. 7:**
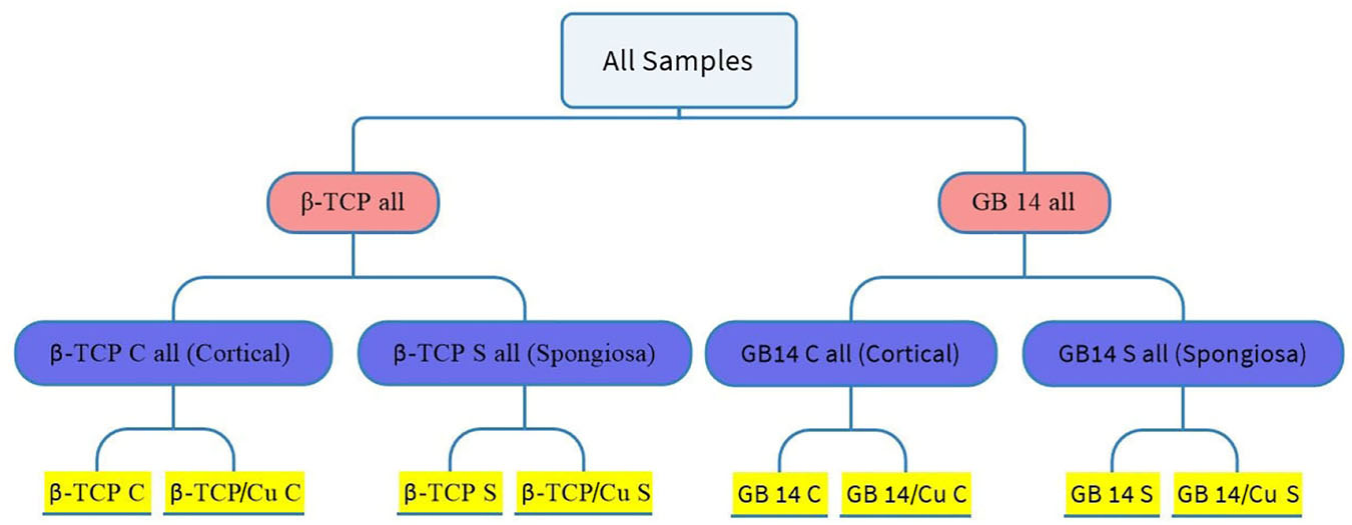
Schematic representation of all experimental sample cohorts. Red: All β-TCP or GB 14 samples; Blue: stratified by position in the bone; Yellow: further stratified by coating type

A total of 12 rabbits were operated on. One from the 12 rabbits was excluded due to acute wound infection. For the evaluation, samples from the push-out test were categorized into four groups according to their coating: β-tricalcium phosphate (β-TCP) and GB14, each with and without copper (Cu) incorporation. Based on their anatomical placement within the bone, the specimens were further subdivided into cortical (C) and spongiosa (S) groups (Fig. 7).

#### β-TCP

**Fig. 8:**
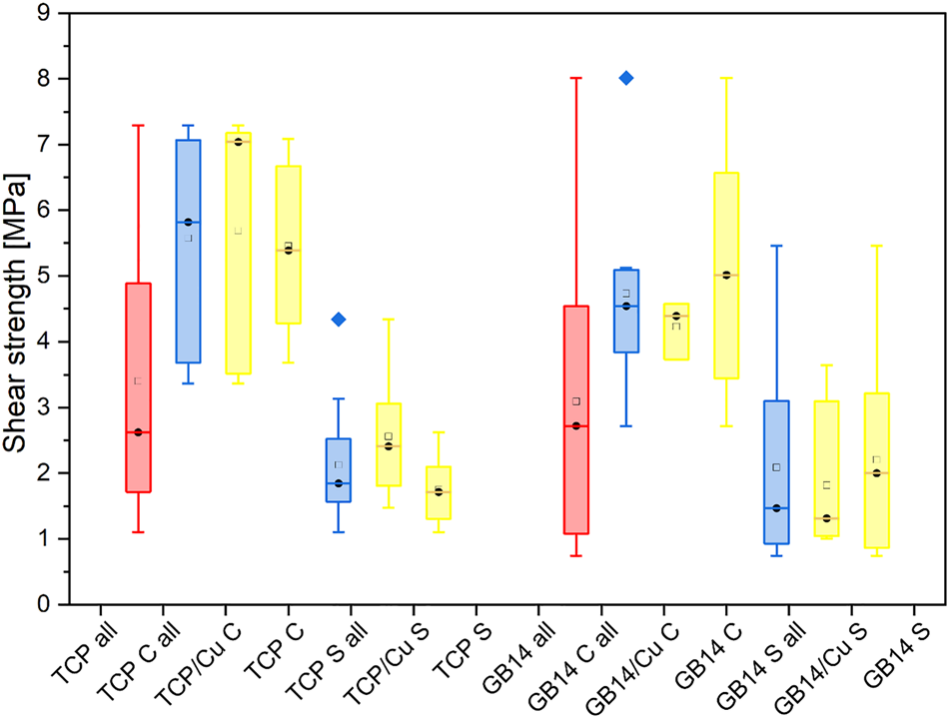
Shear strength of coatings with and without Cu ions after 24 weeks. Red: All β-TCP or GB 14 samples; Blue: stratified by position in the bone; Yellow: further stratified by coating type

The β-TCP group (TCP all), comprising all four β-TCP subgroups (β-TCP/Cu C, β-TCP C, β-TCP/Cu S, β-TCP S), exhibited a mean shear strength of 3.40 MPa (95% CI ± 2.04). Given the significantly higher values observed in cortical compared to spongious samples, the data were further stratified accordingly. The β-TCP cortical group (TCP C all) demonstrated a mean shear strength of 5.57 MPa (95% CI ± 1.59), whereas the spongiosa group reached only 2.13 MPa (95% CI ± 0.81). To assess the influence of Cu incorporation, the cortical β-TCP samples were further separated into those with Cu (TCP/Cu C: 5.68 MPa, 95% CI ± 1.99) and without Cu (TCP C: 5.46 MPa, 95% CI ± 1.30), revealing no significant difference. However, in the spongiosa samples, a statistically significant difference was observed: Cu-doped β-TCP (β-TCP/Cu S) exhibited a higher shear strength of 2.56 MPa (95% CI ± 0.90) compared to Cu-free β-TCP (β-TCP S: 1.75 MPa, 95% CI ± 0.50) (Fig. 8)

#### GB 14

The analysis of the GB14-coated samples was conducted analogously to the β-TCP group. The overall GB14 group (GB14 all) demonstrated a mean shear strength of 3.09 MPa (95% CI ± 1.96). Given the notably higher shear strength in cortical compared to spongiosa samples, the data were further stratified by anatomical location. The cortical subgroup (GB14 C all) exhibited a mean shear strength of 4.73 MPa (95% CI ± 1.53), whereas the spongiosa subgroup (GB14 S all) showed a lower mean of 2.09 MPa (95% CI ± 1.47). Further differentiation based on Cu incorporation revealed the following results: GB14Cu C: 4.27 MPa (95% CI ± 0.47); GB14 C: 5.01 MPa (95% CI ± 1.94); GB14Cu S: 1.82 MPa (95% CI ± 1.23); and GB14 S: 2.21 MPa (95% CI ± 1.62). (Fig. 8)

### Descriptive evaluation of the association between shear strength and relative distance to the implant site

**Fig. 9:**
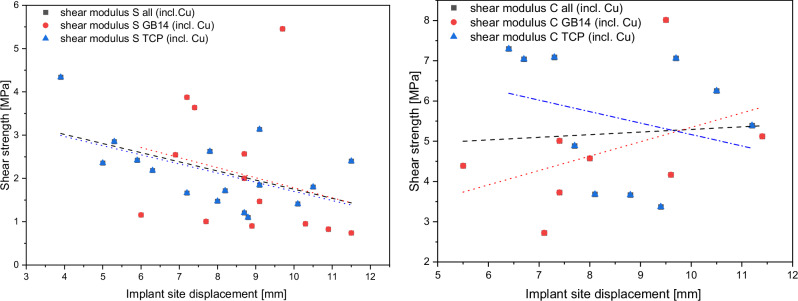
Descriptive analysis of the relationship between shear strength and implant site displacement in spongiosa and cortical bone, with subgroup stratification by coating material. All groups also contain samples with Cu due to an insignificant difference

A descriptive correlation analysis was performed to investigate the relationship between shear strength and displacement of the implant site. For this purpose, computed tomography (CT) image data of the bone around the implant sites was analyzed and correlated with the mechanical measurements. Given the intrinsic heterogeneity in bone composition, we stratified the analysis based on tissue type, distinguishing between cortical bone and spongiosa (trabecular bone). The observed data indicated an inverse relationship between implant displacement and shear strength in the spongiosa. While this inverse association reached statistical significance only within the spongiosa and β-TCP spongiosa categories, a similar pattern was also noted - albeit non-significantly - in both the β-TCP cortical and the GB14 spongiosa subgroups. In contrast, the GB14 cortical subgroup demonstrated a positive correlation between implant displacement and shear strength; however, this trend did not achieve statistical significance. These findings suggest that the mechanical integration of implant materials with trabecular bone may be more sensitive to variations in shear strength than integration with cortical bone (Fig. 9).

## Discussion

In the coming years, the incidence of joint replacement surgeries is expected to increase, in part due to the aging population. As the average age of patients rises, a corresponding increase in the rate of postoperative complications was anticipated [34, 35]. Primary total hip arthroplasty (THA) and total knee arthroplasty (TKA) represent the most commonly performed orthopedic procedures. Recent epidemiological data indicate a proportional increase in postoperative infection rates corresponding with the rising volume of these surgeries [36]. HA remains the most commonly used coating for orthopedic implants today. In the short term, HA-coated implants demonstrate clinical outcomes comparable to cemented prostheses and superior to uncoated implants [37–39]. However, long-term studies indicate that HA coating does not significantly influence implant survival over extended periods [40, 41]. It has been proposed that advancements in implant surface design may diminish the additive benefits of HA coating on long-term outcomes [41]. Despite this, current evidence suggests that HA coating offers clear advantages during the early postoperative phase by promoting osseointegration, enhancing bone ingrowth, and reducing the interface gap between the bone and the implant [38]. A notable concern is that HA is not fully resorbable [17]; particulate debris from the coating may detach, potentially inciting local inflammatory responses or joint inflammation, which could contribute to osteolysis [42, 43]. These findings suggest the necessity for a faster- and fully resorbing coating than HA, in order to reduce undesirable complications while preserving its beneficial properties. The primary focus is on optimizing the short-term advantages of the coating, particularly during the critical period between primary and secondary stabilization. However, such improvements may also influence long-term outcomes. In this study, we focused on two materials - GB14 and β-TCP - which have promising results compared to HA in previous investigations [9]. Our primary objective was to optimize the porosity of the coating in order to enhance the synchronization between its degradation rate and the progression of bone ingrowth. Additionally, we incorporated Cu into the coating using a novel supraparticle-based method, aiming to improve resistance against bacterial biofilm formation on the prosthetic surface [7, 26, 28]. While earlier studies have reported that Cu can adversely affect the osseointegration of HA-coated implants, such negative effects were not observed with GB14 or β-TCP [9]. Moreover, both GB14 [17] and β-TCP [44] exhibit significantly higher resorption rates than HA, which further supports their selection as the focus of this work. In this study, we investigated the in vivo performance of a newly developed, highly porous coating in which Cu was incorporated via superparticles [25]. We assessed the shear strength of implants with either GB14 or β-TCP coatings after 24 weeks of implantation. Due to statistically significant differences observed in certain groups, we stratified the analysis by implant position within the bone (cortical vs. spongiosa) and by Cu-content. Our results consistently demonstrated that samples implanted in cortical bone exhibited significantly higher shear strength compared to those in spongiosa, independent of coating type or Cu-content. No significant differences in shear strength were found between the β-TCP- and GB14-coated implants across the overall sample or within any stratified subgroup. Interestingly, within the spongiosa subgroup, the β-TCP implants containing Cu showed significantly greater shear strength than those without Cu - an effect contrary to what has been previously observed with Cu-incorporated HA coatings. This suggests that not only the presence of Cu, but also the method of incorporation via superparticles, may contribute to enhanced osseointegration of the coating.

We compared our findings with previous studies conducted under similar experimental conditions and uncoated implant surface, as summarized in Table 1 [9, 45]. Over time, we observed a continuous improvement in the osseointegration performance of the implant. A major challenge in comparing results across studies was the differing primary objectives and methodologies. During the course of our investigation, several novel observations were made. Notably, cortical bone samples consistently exhibited significantly higher shear force values than spongiosa samples—an aspect not considered in the study by Przybilla et al. [9].

**Table 1:** A comparison of shear strength (MPa) among the different materials was conducted between the present study and previous investigations performed under comparable experimental conditions

	Shear force [MPa]		
Coating material	Present study	Przybilla et al. [9]	Bernstein [45]
β-TCP	3.40 (95% CI ± 2.04)	3.30 (95% CI, 1.60–5.01)	
β-TCP cort	5.57 (95% CI ± 1.59)		
β-TCP spon	2.13 (95% CI ± 0.81)		
GB14	3.09 (95% CI ± 1.96)	5.47 (95% CI, 4.52–6.43)	2.12 ± 1.01
GB14 cort	4.73 (95% CI ± 1.53)		2.91 ± 0.95
GB14 spon	2.09 (95% CI ± 1.47)		1.68 ± 0.77
HA		2.84 (95% CI, 2.05–3.62)	2.17 ± 1.60
HA cort			3.95 ± 1.16
HA spong			1.13 ± 0.54

A limitation in the comparative analysis arises from the disproportionate representation of cortical and spongiosa samples across studies. In our dataset, the cortical-to-spongiosa sample ratio was not maintained at 1:2, emphasizing the importance of analyzing these groups separately. Another key observation, illustrated in Fig. 5 is the influence of the implantation site’s distance from the femoral condyles. Increased distance from the condyles was associated with reduced shear forces for the spongiosa samples, indicating location-dependent variability in implant stability. For baseline comparison, we refer to the HA-coated samples from the study by Bernstein et al. [45]. Our samples consistently showed higher shear force values, with the sole exception of the GB14 sample, which exhibited higher shear force in previous projects (Przybilla et al. [9]). Nevertheless, it can be assumed that the osseointegration performance of the Cu-doped β-TCP samples in particular was superior to the comparison samples from previous studies (Bernstein [45] and Przybilla [9]), especially the HA samples (Table 1) as these are still the gold standard for implant coatings.

In addition to the significant findings, several non-significant trends of potential interest were observed. A likely explanation for the lack of statistical significance is the limited sample size. In accordance with the 3Rs principle (Replacement, Reduction, and Refinement) governing animal experimentation, we aimed to minimize the number of animals used. To this end, baseline values for HA were adopted from the work of Bernstein et al. [45]. thereby reducing the need for additional experimental animals. Another general limitation in the field is the lack of standardization in the evaluation of novel coating materials. In our study, we employed the New Zealand White rabbit model, which is commonly used for assessing orthopedic coatings. However, there is considerable variability in the literature regarding the characteristics of the uncoated implants. Notably, the surface topography of the uncoated implant significantly influences osseointegration outcomes. Additional critical parameters that vary across studies include implant diameter and implantation site, both of which can substantially affect experimental results.

## Conclusion

In this study, we compared two novel implant coatings: β-Tricalcium Phosphate (β-TCP) and GB-14 that exhibit physicochemical characteristics similar to hydroxyapatite (HA), while offering improved resorption and enhanced osseointegration. Porosity was carefully optimized, and copper was incorporated into the coatings in a manner that preserves their osteoinductive and osteoconductive properties. Copper, as a non-antibiotic antibacterial agent, demonstrated the capacity to reduce infection risk. While it negatively affected the osseointegration of HA, this adverse effect was not observed with β-TCP and GB-14. These findings address two critical complications leading to revision joint arthroplasty: infection and inadequate osseointegration. As the field of joint replacement continues to evolve, with increasing complexity and patient demands, the development and refinement of implant coatings will become ever more important. This underscores the need for standardized testing protocols and greater consistency in evaluating coating performance across studies. Among the various materials tested, β-TCP and GB-14 emerged as particularly promising due to their favorable biological response in the early postoperative phase. However, further investigations with larger sample sizes are necessary to validate these findings. Based on current evidence, both coatings demonstrate substantial potential for future clinical application in joint arthroplasty.

## Data Availability

The raw data supporting the conclusions of this article will be made available by the authors, without undue reservation.
